# Simultaneous Enantiomeric Separation of Carfentrazone-Ethyl Herbicide and Its Hydrolysis Metabolite Carfentrazone by Cyclodextrin Electrokinetic Chromatography. Analysis of Agrochemical Products and a Degradation Study

**DOI:** 10.3390/molecules26175350

**Published:** 2021-09-02

**Authors:** Laura García-Cansino, María Ángeles García, María Luisa Marina

**Affiliations:** 1 Universidad de Alcalá, Departamento de Química Analítica, Química Física e Ingeniería Química, Ctra, Madrid-Barcelona Km, 33.600, 28871 Alcalá de Henares (Madrid), Spain; laura.garciacansino@uah.es (L.G.-C.); angeles.garcia@uah.es (M.Á.G.); 2 Universidad de Alcalá, Instituto de Investigación Química Andrés M, del Río, Ctra, Madrid-Barcelona Km, 33.600, 28871 Alcalá de Henares (Madrid), Spain

**Keywords:** carfentrazone-ethyl, carfentrazone, cyclodextrin electrokinetic chromatography, chiral separation, commercial herbicide formulations, degradation study

## Abstract

The different activity and toxicity that the enantiomers of agrochemicals may have requires the development of stereoselective analytical methodologies enabling the individual determination of each enantiomer. The aim of this work was to develop the first Electrokinetic Chromatography methodology enabling the simultaneous enantiomeric separation of carfentrazone-ethyl herbicide and its hydrolysis metabolite carfentrazone. The use of an anionic cyclodextrin as chiral selector (captisol at 2.5% (*w/v*)) in a 25 mM acetate buffer, at a temperature of 30 °C, and an applied voltage (reverse polarity) of −30 kV, allowed the simultaneous separation of the four enantiomers of the two compounds studied in 6.8 min with enantiomeric resolutions of 5.0 for carfentrazone-ethyl and 5.1 for carfentrazone. Analytical characteristics of the developed method were evaluated and found adequate to achieve the quantitation of carfentrazone-ethyl and carfentrazone. Analysis of a commercial herbicide formulation showed the potential of the method for the quality control of these agrochemical products. Degradation studies for carfentrazone-ethyl revealed that no significant degradation took place in cleaned sand samples while a significant but not stereoselective degradation took place in soils for the whole period of time considered (seven days).

## 1. Introduction

Approximately 30–40% of agrochemicals have at least one chiral center in their molecule [1] and are usually marketed as racemates [2]. However, their enantiomers may have a different activity or environmental behavior so chiral analytical methodologies are required in order to achieve the quality control of commercial agrochemical formulations or the analysis of environmental samples. In fact, these chiral agrochemicals can be degraded into other related compounds after application, which may or may not be chiral, and which may not have the same activity or may have a higher toxicity [3]. Therefore, it is also necessary to carry out degradation studies of chiral agrochemicals applied to soils to check the appearance of other compounds and to know how they degrade. 

Carfentrazone-ethyl (ethyl-(*R*,*S*)-2-chloro-3-(2-chloro-5-(4-difluoromethyl-4,5-dihydro-3-methyl-5-oxo-1H-1,2,4-triazol-1-yl)-4-fluorophenyl) propionate) is a chiral postemergence triazole herbicide used against broadleaf weeds in wheat, corn, and soybeans [4]. It is commercialized as a racemic mixture although Duan et al. demonstrated that the S-enantiomer presented double herbicidal activity and 4.8 times more toxicity towards aquatic organisms than (*R*)-carfentrazone-ethyl [5]. Hydrolysis of this compound originates its free acid metabolite, carfentrazone ((*R*,*S*)-2-chloro-3-(2-chloro-5-(4-difluoromethyl-4,5-dihydro-3-methyl-5-oxo-1H-1,2,4-triazol-1-yl)-4 fluorophenyl) propionic acid), which is also chiral [6]. This metabolite also exhibits herbicidal activity but, unlike carfentrazone-ethyl, there is differential tolerance between species due to its breakdown into unknown metabolites [7].

According to the literature, the enantiomeric separation of carfentrazone-ethyl has been carried out by HPLC with UV or MS detection. Using UV detection, values obtained for the enantiomeric resolution for this herbicide ranged from 0.61 to 3.42 in analysis times from 10 to 30 min [8,9,10,11,12,13], while with MS/MS detection enantiomeric resolutions close to 4.5 in 4 min [5] and 1.5 in 35 min [14] were obtained. The individual enantiomeric separation of carfentrazone-ethyl, carfentrazone and ninety-eight different pesticides was described by HPLC-UV, with resolution values of 2.0 and 2.5, and analysis times of 3.2 min and 7.5 min, for carfentrazone-ethyl and carfentrazone, respectively, at a temperature of 25 °C [15]. 

In two other works carried out by Duan et al., the simultaneous enantiomeric separation of both carfentrazone-ethyl and its free acid metabolite (carfentrazone) was reported, and each of the four enantiomers was identified. In the first work, they performed the simultaneous chiral separation of both compounds in 20 min by HPLC and with enantiomeric resolutions of 1.0 for carfentrazone and 3.2 for carfentrazone-ethyl [16]. In the second one, they studied the degradation of carfentrazone-ethyl and its metabolite in three different soils [17] using UPLC as the analytical separation technique (analysis time was 22.5 min). They concluded that *R*-(+)-carfentrazone-ethyl and *S*-(+)-carfentrazone were preferentially degraded, while the corresponding enantiomers were enriched. In both works they used MS/MS as a detector and worked at a temperature of 35 °C [16,17].

Although HPLC has been the most employed technique for chiral separations and its implementation at an industrial level exceeds that reached by other chiral separation techniques [18], capillary electrophoresis (CE) has shown to be a powerful analytical technique to achieve chiral separations. Compared to HPLC, CE has the advantage of using minimal volumes of reagents and solvents, as well as not requiring the use of a chiral separation column (which is very often employed in HPLC), thus reducing costs [19]. The most used CE mode for chiral separations is Electrokinetic Chromatography (EKC), which only requires the addition of chiral selectors (in most cases usually cyclodextrins (CDs)) in the separation medium to obtain enantiomeric separations [20]. The most widely accepted recognition mechanism for the enantioseparation of a chiral analyte (“guest”) with a CD (“host”) is based on the formation of inclusion complexes in which the chiral analyte is included into the CD cavity through bonds with the secondary hydroxyl groups located on the glucopyranose ring that forms the CD, generating the so-called “host-guest” inclusion complex. However, the recognition mechanism is not always based on the formation of such complexes, but partial inclusion or external intermolecular interactions may be sufficient [18].

The aim of this work was to develop the first EKC methodology enabling the simultaneous enantiomeric separation of carfentrazone-ethyl and its hydrolysis metabolite carfentrazone and to apply it to the analysis of commercial herbicide formulations and to degradation studies of these compounds in environmental samples (sand and soil).

## 2. Results

### 2.1. Development of a Chiral CD-EKC Methodology for the Simultaneous Separation of Carfentrazone-Ethyl and Carfentrazone

**Figure 1:**
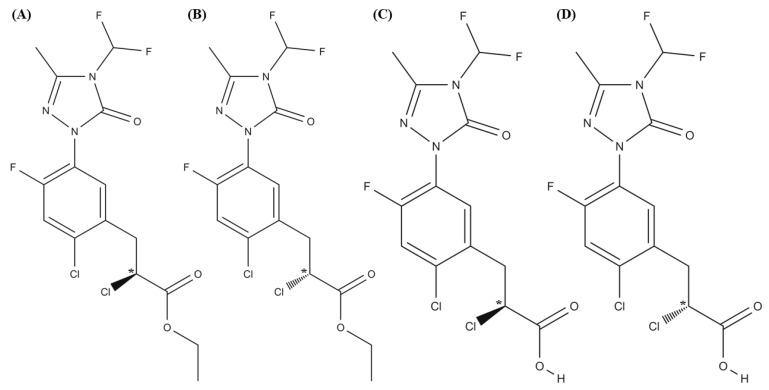
Structures of (**A**,**B**) carfentrazone-ethyl enantiomers; (**C**,**D**) carfentrazone enantiomers.

As reported in the literature [21], carfentrazone-ethyl is stable at pH 5.0 while it hydrolyzes at pH 7.0 in 8.6 days and at pH 9.0 in 3.6 h originating its free acid (carfentrazone) (see Figure 1). 

Both compounds are chiral possessing each a pair of enantiomers. Their enantiomeric determination and the study of the degradation of carfentrazone-ethyl herbicide at an enantiomeric level requires the development of methodologies enabling the simultaneous enantiomeric separation of both compounds. With this aim, a screening was carried out with ten anionic CDs (carboxymethyl-β-CD (CM-β-CD), sulphated-β-CD (S-β-CD), carboxyethyl-β-CD (CE-β-CD), carboxyethyl-γ-CD (CE-γ-CD), carboxymethyl-γ-CD (CM-γ-CD), succinyl-β-CD (Succ-β-CD), succinyl-γ-CD (Succ-γ-CD), sulphated-γ-CD (S-γ-CD), phosphated-β-CD (Ph-β-CD), sulfobutylether-β-CD (captisol)) using a 100 mM sodium acetate buffer (pH 5.0) at a temperature of 20 °C and an applied voltage of −20 kV (negative polarity was employed to short analysis times since studied compounds were neutral and CDs were anionic). pH 5.0 was selected since carfentrazone-ethyl is stable at this pH value as mentioned above. Three out of ten CDs employed (CM-β-CD, Ph-β-CD, and captisol) showed chiral discrimination against both studied compounds. However, taking into account the analysis times obtained (49.0, 55.0 and 14.8 min, respectively), captisol was chosen as the best chiral selector. 

**Figure 2:**
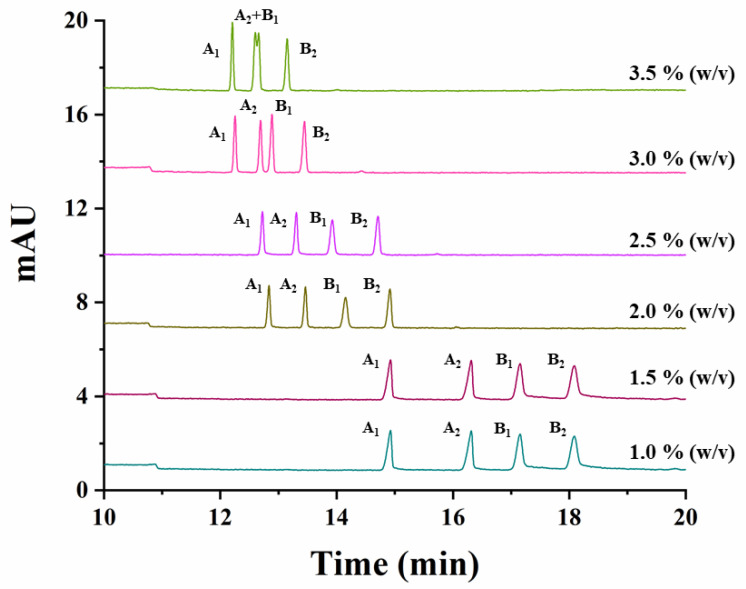
Electropherograms showing the effect of captisol concentration on the simultaneous separation of carfentrazone-ethyl and carfentrazone enantiomers using a standard solution containing 30 mg L^−1^ carfentrazone-ethyl racemate and 20 mg L^−1^ carfentrazone racemate with a 100 mM sodium acetate buffer (pH 5.0). Experimental conditions: uncoated fused-silica capillary, 58.5 cm (50 cm effective length × 50 µm i.d.); hydrodynamic injection: 50 mbar × 10 s; temperature: 20 °C; voltage: −20 kV; λ: 245 nm ± 4 nm. A_1_ and A_2_: carfentrazone-ethyl enantiomers. B_1_ and B_2_: carfentrazone enantiomers.

The effect of the variation of the CD concentration, the temperature, the buffer concentration and the separation voltage, on the simultaneous separation of both studied compounds, was investigated. The effect of the CD concentration was studied by varying the percentage of captisol from 1% to 3.5% (*w/v*) (1.0%, 1.5%, 2.0%, 2.5%, 3.0%, 3.5%). Figure 2 shows that a decrease in the resolution for carfentrazone-ethyl as well as in the analysis time for both compounds was observed when increasing the percentage of captisol in the separation medium. A 2.5% (*w/v*) captisol was the selected CD percentage for further experiments taking into account the resolutions and analysis time obtained under these conditions. 

**Figure 3:**
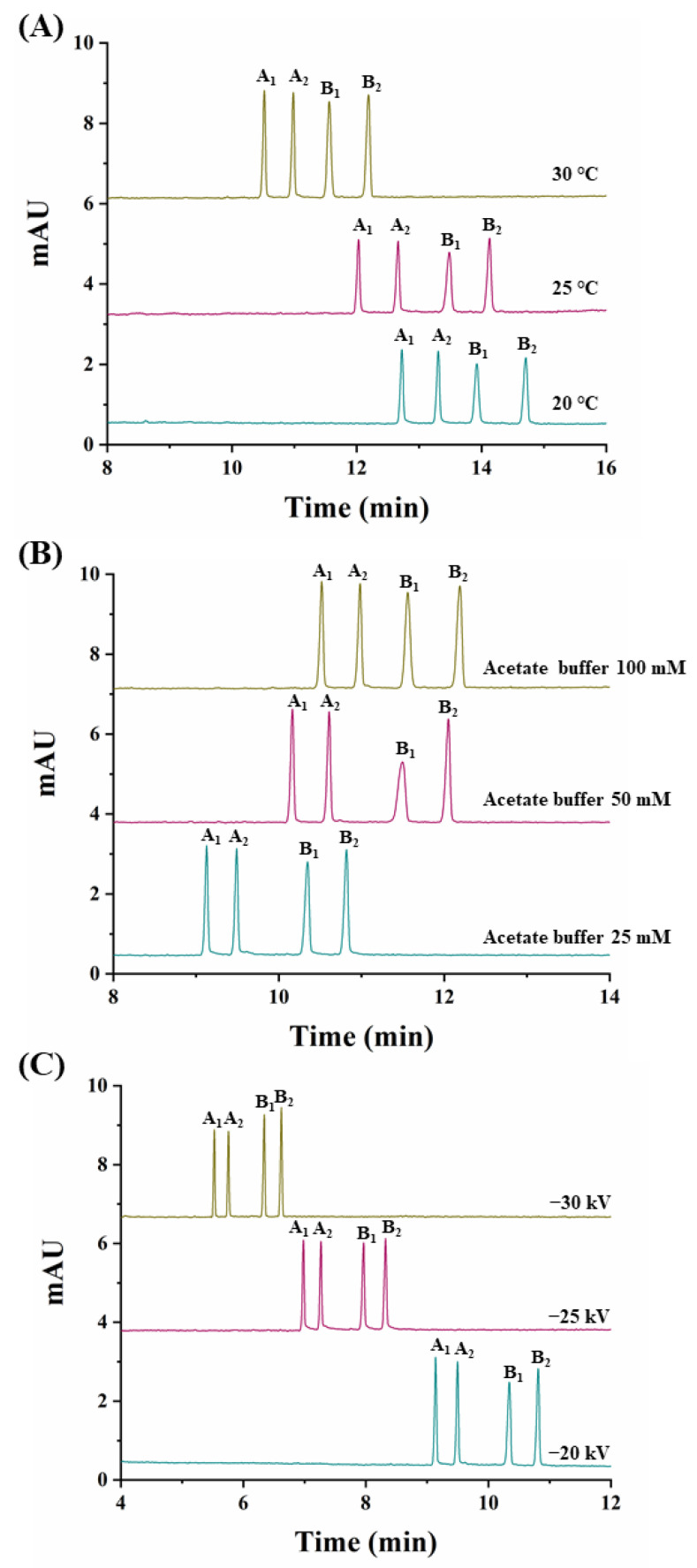
Electropherograms corresponding to the simultaneous separation of carfentrazone-ethyl and carfentrazone enantiomers from a standard solution containing 30 mg L^−1^ of carfentrazone-ethyl racemate and 20 mg L^−1^ of carfentrazone racemate. (**A**) Effect of the temperature; (**B**) Effect of the sodium acetate buffer concentration; (**C**) Effect of the applied voltage. Other experimental conditions as in Figure 2. A_1_ and A_2_: carfentrazone-ethyl enantiomers. B_1_ and B_2_: carfentrazone enantiomers.

At this CD concentration, the temperature was varied (20 °C, 25 °C, and 30 °C) (Figure 3A). A value of 30 °C was chosen as it allowed the shortest analysis time (12.3 min) and the highest peak areas (see Table S1 in Supplementary Material).

Previous to the study of the effect of the applied voltage and taking into account that with a 100 mM buffer concentration, a current of 73 µA was obtained, the buffer concentration was optimized. Using concentrations of sodium acetate buffer of 25 mM and 50 mM, currents decreased to 42 and 52 µA, respectively. Moreover, as shown in Figure 3B, at a 25 mM buffer concentration, analysis times decreased without a loss in resolution so this value was selected as optimum. Under these conditions, the effect of the applied voltage was investigated (−20 kV, −25 kV, and −30 kV). Figure 3C shows that at a −30 kV value, analysis times were the lowest with good resolutions (see Table S1 in Supplementary Material) and acceptable current values of 75 µA. 

Under the optimized conditions (2.5% (*w/v*) captisol, 30 °C, 25 mM acetate buffer, −30 kV), the simultaneous enantiomeric separation of carfentrazone-ethyl and carfentrazone was achieved in 6.8 min with enantiomeric resolutions of 5.0 for carfentrazone-ethyl and 5.1 for carfentrazone (Figure 3C). 

### 2.2. Analytical Characteristics of the Developed Method 

In order to apply the developed method to the chiral determination of carfentrazone-ethyl and carfentrazone, its analytical characteristics were evaluated in terms of linearity, precision, accuracy, and limits of detection (LOD) and quantitation (LOQ) (Table 1).

The linearity was determined using nine standard solutions of carfentrazone-ethyl racemate and nine standard solutions of carfentrazone racemate, both at different concentration levels ranging from 0.6 to 100.0 mg L^−1^. Results were adequate with R^2^ values greater than 99% for each enantiomer.

Precision of the method was evaluated considering instrumental repeatability, method repeatability and intermediate precision for peak areas and migration times for the two pairs of enantiomers. Two concentration levels for each racemate (10 mg L^−1^ and 60 mg L^−1^) were employed.

Instrumental repeatability was determined from six repeated injections of each one of the above standard solutions, resulting RSD values (%) lower than 0.7 and 1.0% for migration times and corrected peak areas, respectively, to the low level of concentration, and RSD values (%) lower than 1.0 and 1.1% for migration times and corrected peak areas, respectively, to the high level of concentration. Method repeatability was evaluated by injecting three replicates three times on the same day of carfentrazone-ethyl and carfentrazone standard solutions. RSD values (%) were lower than 1.3 and 1.9% for migration times and corrected peak areas, respectively, to the low level of concentration, and lower than 1.2 and 2.0% for migration times and corrected peak areas, respectively, to the high level of concentration. Intermediate precision was obtained by injecting in triplicate three replicates of carfentrazone-ethyl and carfentrazone standard solutions during three consecutive days. RSD values (%) lower than 1.3 and 2.1% for migration times and corrected peak areas, were obtained, respectively, to the low level of concentration, and lower than 1.5 and 2.5% for migration times and corrected peak areas, respectively, to the high level of concentration (Table 1). 

Limits of detection (LOD) and quantitation (LOQ) obtained theoretically for the four enantiomers (LOD = 3.29 × S_intercept_/slope and LOQ = 10 × S_intercept_/slope) were 1.03 and 3.12 mg L^−1^ for A_1_ carfentrazone-ethyl, respectively, 1.04 and 3.17 mg L^−1^ for A_2_ carfentrazone-ethyl, respectively, and 0.72 and 2.20 mg L^−1^ for both carfentrazone enantiomers (B_1_ and B_2_), respectively. These values were higher than those experimentally observed considering a signal to noise ratio (S/N) of 3 for LOD and 10 for LOQ (0.4 mg L^−1^ for LOD and 1.4 mg L^−1^ for LOQ for both carfentrazone-ethyl enantiomers, and 0.3 mg L^−1^ for LOD and 0.8 mg L^−1^ and 0.9 mg L^−1^ for LOQ for B_1_ and B_2_, respectively, of carfentrazone) (Table 1).

### 2.3. Determination of Carfentrazone-Ethyl in a Commercial Herbicide Formulation

**Figure 4:**
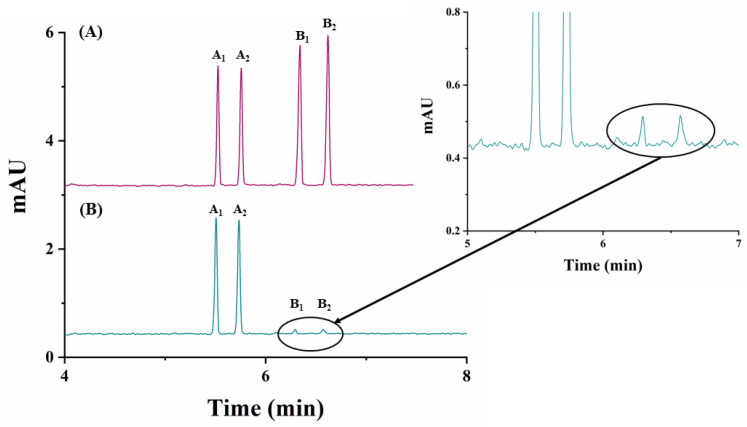
Electropherograms corresponding to the analysis of a carfentrazone-ethyl-based commercial agrochemical formulation using 25 mM sodium acetate buffer (pH 5.0)/2.5% (*w/v*) captisol, 30 °C and −30 kV. (**A**) Standard solution containing 30 mg L^−1^ of carfentrazone-ethyl racemate and 20 mg L^−1^ of carfentrazone racemate; (**B**) commercial herbicide formulation containing 30 mg L^−1^ of carfentrazone-ethyl racemate according to the label. Other experimental conditions as in Figure 2. A_1_ and A_2_: carfentrazone-ethyl enantiomers. B_1_ and B_2_: carfentrazone enantiomers.

The analytical method developed was applied to the quantitation of carfentrazone-ethyl enantiomers in a commercial herbicide formulation. Figure 4B shows the electropherogram corresponding to the enantiomeric separation of carfentrazone-ethyl in the commercial agrochemical formulation. 

As it can be seen in this figure, the analytical method developed shows good selectivity, without any interfering peaks from possible matrix components, but two analytical signals corresponding to the pair of enantiomers of carfentrazone appeared. Each of the enantiomers corresponding to carfentrazone are present in the commercial herbicide formulation at a concentration below their LOQ, so they cannot be quantified, but they can be detected (estimated concentrations were 0.6 mg L^−1^ and 0.8 mg L^−1^ for B_1_ and B_2_ enantiomers, respectively).

To investigate the existence of matrix interferences, a comparison of slopes obtained by the external standard and the standard additions calibration methods for the commercial herbicide formulation was carried out. For this purpose, five known amounts of carfentrazone-ethyl racemate were added to the commercial herbicide formulation samples containing a constant amount of carfentrazone-ethyl. As shown in Table 1, the slope values did not show significant differences, so it could be concluded that there were no matrix interferences (*p*-values > 0.05 for a 95% confidence level) and the external standard calibration method can be applied to quantify the content of carfentrazone-ethyl in the commercial herbicide formulation.

The accuracy of the method was assessed for both carfentrazone-ethyl enantiomers as the recovery (in percentage) obtained when six solutions of the commercial herbicide formulation containing 15 mg L^−1^ of each enantiomer (according to the label) were spiked with 10 mg L^−1^ of carfentrazone-ethyl standard racemate and other six solutions of the commercial formulation at the same enantiomer concentration were spiked with 60 mg L^−1^ of carfentrazone-ethyl standard racemate. Recovery values determined for carfentrazone-ethyl racemate in the herbicide formulation were 104 ± 4% (104 ± 4% for the A_1_ enantiomer and 104 ± 5% for the A_2_ enantiomer), which included the 100% value. The determined content of the carfentrazone-ethyl racemate in the commercial formulation was 6.2 ± 0.3% (*w/v*) (3.1 ± 0.1% (*w/v*) per enantiomer), which was in agreement with the labelled content (6% (*w/v*)).

Although the analyzed commercial formulation was based on the use of the herbicide racemate, there is a tendency to market enantiomerically pure herbicide formulations when the activity of both enantiomers differs, as in this case. So, as many herbicides commercial formulations need a re-evaluation, our methodology could be a powerful tool for the quality control of carfentrazone-ethyl based herbicide formulations that could be marketed as pure enantiomer in a future. 

### 2.4. Degradation of Carfentrazone-Ethyl in Sand and Soil Samples

In order to achieve a degradation study of carfentrazone-ethyl in environmental samples (sand and soil), a study on the existence of matrix interferences was carried out for both carfentrazone-ethyl and carfentrazone as its hydrolysis metabolite. The comparison of the slopes obtained for the enantiomers of both compounds by the external and the standard additions methods for sand and soil samples showed that there were not matrix interferences (Table 1) so the external calibration method could be employed to achieve their quantitation in environmental samples and to evaluate the extraction yield and method accuracy.

The extraction conditions of both compounds were evaluated using cleaned sand samples individually spiked with each standard racemate solution at a concentration of 20 mg L^−1^. The use of a high intensity ultrasounds probe was compared with magnetic stirring by varying the experimental conditions (amplitude values for high-intensity ultrasounds were 20, 30, and 60% and extraction times were 3, 5, and 10 min; magnetic stirring was applied for 5, 10, and 15 min). Extraction yields varied from 50 to 96% under the different experimental conditions. Results showed that the use of magnetic stirring gave rise to the highest extraction percentage for the four enantiomers of both compounds originating an average extraction percentage of 96.0%. Therefore, these extraction conditions were selected as optimal to achieve the degradation study for both compounds in sand and soils over seven days. 

The accuracy of the method was assessed for both carfentrazone-ethyl and carfentrazone enantiomers as the recovery (in percentage) obtained when thirteen extracts (seven from cleaned sand samples and six from soil samples) were spiked at different racemate concentration levels (from 0.6 to 100 mg L^−1^) (each one injected in triplicate). Recovery values for every enantiomer ranged from 94 to 98% in sand samples and from 97 to 105% for soil samples (see Table 1). 

**Figure 5:**
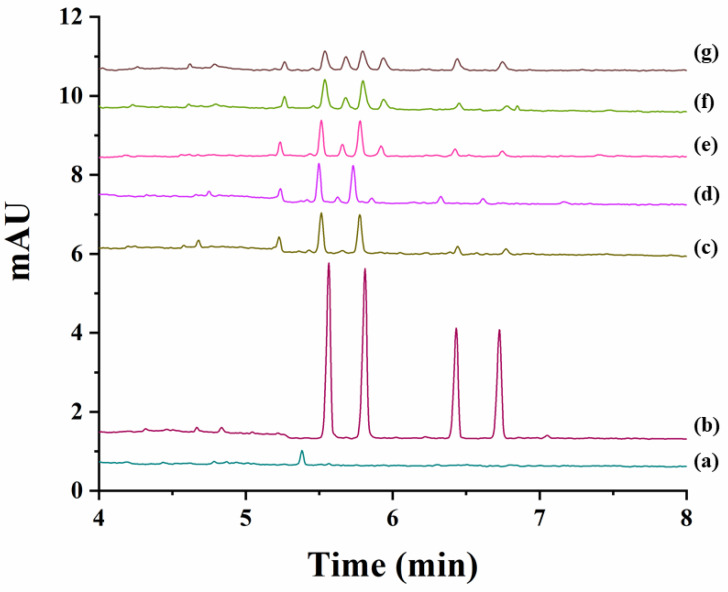
Electropherograms corresponding to the analysis of the extracts obtained from soil samples spiked with 40 mg L^−1^ carfentrazone-ethyl racemate (degradation study of carfentrazone-ethyl in soil samples). (**a**) Soil extract blank; (**b**) Standard solution of carfentrazone-ethyl racemate and carfentrazone racemate at 40 mg L^−1^ and 20 mg L^−1^, respectively; (**c**) soil extract after zero days; (**d**) soil extract after one day; (**e**) soil extract after three days; (**f**) soil extract after four days; (**g**) soil extract after seven days.

The degradation study was next individually achieved for carfentrazone-ethyl and carfentrazone in sand and soil samples by spiking them with 40 mg L^−1^ carfentrazone-ethyl or 20 mg L^−1^ carfentrazone racemates. Results are shown in Figures S1 and S2 for sand and soil samples, respectively. No significant degradation was observed for both compounds in sand samples (Figure S1) nor for carfentrazone in soil samples although in this case degradation could reach a 15% at the seventh day (Figure S2B). A very different situation was observed in the case of carfentrazone-ethyl in soil samples for which a degradation close to 70% was observed from zero to four days (reaching an 80% degradation at the seventh day) (Figure S2A). Figure 5 shows the electropherograms corresponding to the analysis of the soil extracts spiked with 40 mg L^−1^ carfentrazone-ethyl racemate during the period of seven days considered. No stereoselective degradation was observed. However, two new peaks appeared in the electropherograms during the degradation study that could correspond to additional degradation products. Figure 5 also shows that degradation of carfentrazone-ethyl originated the appearance of carfentrazone peaks although to a less extent (around 7% from days 0 to 4 and 9.5% at the seventh day, percentages expressed as concentration of carfentrazone divided by the concentration of degraded carfentrazone-ethyl). The differences in the results obtained for sand (pH 6.4) and for soil (pH 9.0) samples could be due to the pH values for each sample and the same can be said for the non-stereoselective behaviour observed in this work when compared with previous works reporting a stereoselective degradation of carfentrazone-ethyl and carfentrazone in three kinds of soils with pH values ranging from 5.2 to 7.0 [17]. In fact, pH can drastically affect the degradation stereoselectivity for some compounds [22]. All the above-mentioned shows that the results obtained in the present work are complementary to other previously obtained results using other techniques and other soils with different complexity showing the need of further research as stated in previous works [17].

Recovery values obtained for the CE method developed in this work were compared to those reported for HPLC methods. Only four articles studied the analytical characteristics of the methods developed by HPLC for the enantioseparation of carfentrazone-ethyl [5,8,14,16] and carfentrazone [16]. In all these works, accuracy and precision were determined by intra-day and inter-day recovery assays in different matrices (methanol, aeration water, sterile water, and BG11 culture medium [5], soil, water, and wheat [8], soil and water [14], rice plant, wheat plant, corn plant, brown rice, rice husk, wheat, corn, and soil [16]). With this aim, recovery (%) and RSD (%) were evaluated, respectively. Recoveries obtained ranged from 83.2 to 106.5% [5], from 86.3 to 103.3% [8], from 88.2 to 103.1% [14], and from 77.5 to 102.8% [16], similar values to those obtained in the present work. Regarding RSD (%) values corresponding to the above-mentioned recoveries for HPLC methods, they ranged from 1.2 to 11.9% [5], from 3.3 to 7.2% [8], from 4.8 to 8.0% [14], and from 0.4 to 9.8% [16]. In the present work, precision was evaluated for corrected peak areas and migration times and RSD (%) values were lower than 2.5 and 1.5%, respectively.

## 3. Materials and Methods

### 3.1. Reagents and Samples

Glacial acetic acid, and sodium hydroxide were purchased from Sigma-Aldrich (St. Louis, MO, USA). Methanol and hydrochloric acid were from Scharlab S.L. (Barcelona, Spain). The water employed was purified in a Millipore Milli-Q system (Bedford, MA, USA).

The following anionic CDs were assayed as chiral selectors: CM-β-CD (DS 3.0), and S-β-CD (DS 12.0–15.0) from Sigma-Aldrich; CE-β-CD (DS 2.9), CE-γ-CD (DS 3.3), CM-γ-CD (DS 3.5), Succ-β-CD (DS 3.4), Succ-γ-CD (DS 3.5), S-γ-CD (DS 10.0), and Ph-β-CD (DS 4.0) from Cyclolab (Budapest, Hungary); and captisol from Cydex Pharmaceuticals (Lawrence, Kansas).

Carfentrazone-ethyl and carfentrazone were supplied from Sigma-Aldrich and Toronto Canada Research Chemicals, respectively. The commercial herbicide formulation of carfentrazone-ethyl was from FMC Corporation (Philadelphia, PA, USA). Cleaned and dried sand was from Labkem (Barcelona, Spain). Soil used in this work was collected from Villa del Río (Córdoba, Spain). 

### 3.2. Apparatus

Agilent 7100 CE system from Agilent Technologies (Waldbronn, Germany) with a diode array detector (DAD) was used to perform the different electrophoretic experiments. The electrophoretic system was controlled with the HP ^3D^CE ChemStation software that included data collection and analysis. Separations were achieved in uncoated fused-silica capillaries (58.5 cm total length (50 cm effective length) × 50 µm I.D.) from Polymicro Technologies (Phoenix, AZ, USA), at 30 °C in negative-polarity mode. Injections were performed by applying a pressure of 50 mbar for 10 s and using a detection wavelength of 245 nm (band width 4 nm and reference 350 nm). Conditioning of a new capillary was achieved by flushing 30 min sodium hydroxide 1 M, 15 min Milli-Q water and 60 min background electrolyte (BGE). At the beginning of each working day, the capillary was flushed 10 min with 0.1 M sodium hydroxide, 5 min with Milli-Q water, 5 min with 0.1 M HCl and 10 min with buffer solution. Repeatability between injections was ensured with 2 min with 0.1 M sodium hydroxide, 2 min with Milli-Q water, 2 min with 0.1 M HCl, 2 min with buffer solution and 2 min with BGE. 

OHAUS Adventurer Analytical Balance (Nänikon, Switzerland) was used to weight the amounts of the different reagents and standards. pHmeter model 744 from Metrohm (Herisau, Switzerland) was employed for pH measurements. Ultrasonic bath B200 from Branson Ultrasonic Corporation (Danbury, CO, USA) was used for the sonication of all solutions. A High Intensity Focused Ultrasounds (HIFU) probe (model VCX130, Sonics Vibre-Cell, Hartford, CT, USA) was employed in the optimization study on the extraction of the studied compounds from sand samples.

### 3.3. Preparation of Standards and Samples 

Stock standard solutions of carfentrazone-ethyl and carfentrazone racemates (both 2000 mg L^−1^) were prepared by dissolving the appropriate amount of these compounds in MeOH. As carfentrazone-ethyl is a stable compound in solution at pH 5.0 for a long time, standard working solutions at different concentration levels were prepared by appropriate dilution of the stock standard solutions in MeOH (both carfentrazone-ethyl and carfentrazone racemates) dissolving them in the buffer solutions at pH 5.0. 

Commercial formulation contained 6% (*w/v*) carfentrazone-ethyl, so that the necessary amount of this formulation was dissolved in MeOH to obtain a diluted solution of 2000 mg L^−1^, from which the working solutions were prepared at the different desired concentrations diluting with sodium acetate buffer at pH 5.0.

The soil was collected, dried and stored in the dark. The sand was already dried and cleaned. To corroborate that the samples did not contain carfentrazone-ethyl and carfentrazone, they were extracted with 25 mM acetate buffer (pH 5.0), using the optimized extraction method based on 10 min of magnetic stirring and then, centrifugation at 4000× g at 20 °C and for 5 min and injection in CE system. To perform degradation studies of both carfentrazone-ethyl and carfentrazone, 1 g soil and 1 g sand samples were spiked with each of the racemic standards at 40 mg L^−1^ and 20 mg L^−1^, respectively. The mixtures were shaken for 5 min to bring each of the compounds into contact with all soil and sand particles and stored for the required days in the dark. Finally, they were extracted under the optimal extraction method adding a 25 mM acetate buffer (pH 5.0), 10 min of magnetic stirring, and finally, centrifugation at 4000× *g* for 5 min at 20 °C temperature.

All solutions were filtered before use through disposable nylon 0.45 μm pore size filters purchased from Scharlau (Barcelona, Spain).

## 4. Conclusions

Electrokinetic Chromatography enabled the simultaneous enantiomeric separation of carfentrazone-ethyl herbicide and its hydrolysis metabolite carfentrazone using an anionic cyclodextrin as chiral selector. This is the first CE methodology allowing the enantiomeric separation of each of the two compounds studied in this work as well as the simultaneous separation of their four enantiomers. The influence of chiral selector and buffer concentrations, temperature and applied voltage (reverse polarity) were investigated. Under optimized conditions, the simultaneous separation of the four enantiomers of the two compounds studied in 6.8 min with enantiomeric resolutions of 5.0 for carfentrazone-ethyl and 5.1 for carfentrazone. The developed method showed its applicability to the successful quantitation of carfentrazone-ethyl in commercial herbicide formulations and degradation studies in environmental samples. No significant degradation was observed for carfentrazone-ethyl and carfentrazone in cleaned sand samples not for carfentrazone in soil samples (less than 15%). However, significant but not stereoselective degradation (up to 80%) was observed for carfentrazone-ethyl in soil samples during the period of time under study (7 days) giving rise to enrichments of carfentrazone of up to 9.5%. The differences observed with respect to a previous work reporting the stereoselective degradation of carfentrazone-ethyl under other experimental conditions could be justified on the base of the different complexity and pH values of the soil samples under study. Results obtained in this work show the potential of the developed methodology to achieve the quality control of commercial agrochemical formulations and to study the stereoselectivity of degradation processes in environmental samples.

## Figures and Tables

**Table 1:** Analytical characteristics of the CE methodology developed for the simultaneous enantiomeric separation of carfentrazone-ethyl and carfentrazone using captisol as chiral selector.

	Carfentrazone-Ethyl				Carfentrazone			
	A_1_		A_2_		B_1_		B_2_	
**External standard calibration method ^a^**								
**Range**	**0.3–50 mg L^−1^**		**0.3–50 mg L^−1^**		**0.3–50 mg L^−1^**		**0.3–50 mg L^−1^**	
**Slope ± t × S_slope_**	0.064 ± 0.001		0.063 ± 0.002		0.091 ± 0.002		0.091 ± 0.002	
**Intercept ± t × S_intercept_**	−0.040 ± 0.050		−0.050 ± 0.050		−0.030 ± 0.050		−0.050 ± 0.050	
**R^2^**	99.8%		99.8%		99.8%		99.8%	
**Standard additions calibrations method**								
***Commercial formulation*** **^b^**								
**Range**	**0–35 mg L^−1^**		**0–35 mg L^−1^**		--		--	
**Slope ± t × S_slope_**	0.067 ± 0.006		0.066 ± 0.006		--		--	
**R^2^**	99.0%		99.1%		--		--	
***p*** **-value ^c^**	0.6402		0.8569		--		--	
**Accuracy ^d^**								
**Recovery ^1^ (%)**	102 ± 5		99 ± 5		--		--	
**Recovery ^2^ (%)**	101 ± 3		98 ± 3		--		--	
***Sand sample*** **^e^**								
**Range**	**0.3–40 mg L^−1^**		**0.3–40 mg L^−1^**		**0.3–50 mg L^−1^**		**0.3–50 mg L^−1^**	
**Slope ± t × S_slope_**	0.065 ± 0.002		0.063 ± 0.002		0.091 ± 0.003		0.091 ± 0.006	
**R^2^**	99.9%		99.9%		99.9%		99.4%	
***p*** **-value ^c^**	0.5370		0.5114		0.9500		0.7009	
**Accuracy ^f^**								
**Recovery (%)**	98 ± 7		95 ± 7		94 ± 6		95 ± 5	
***Soil sample*** **^g^**								
**Range**	**0.3–40 mg L^−1^**		**0.3–40 mg L^−1^**		**0.3–40 mg L^−1^**		**0.3–40 mg L^−1^**	
**Slope ± t × S_slope_**	0.062 ± 0.003		0.062 ± 0.003		0.089 ± 0.002		0.089 ± 0.002	
**R^2^**	99.8%		99.8%		99.9%		99.9%	
***p*** **-value ^c^**	0.1169		0.2470		0.1784		0.1362	
**Accuracy ^h^**								
**Recovery (%)**	104 ± 5		105 ± 5		97 ± 4		98 ± 4	
**Precision**								
**mg L^−1^**	**5**	**30**	**5**	**30**	**5**	**30**	**5**	**30**
***Instrumental repeatability*** **^i^**								
**t, RSD (%)**	0.7	0.9	0.6	0.9	0.7	0.9	0.7	1.0
**A_c_, RSD (%)**	1.0	1.1	1.0	1.0	0.9	1.1	0.9	1.0
***Method repeatability*** **^j^**								
**t, RSD (%)**	1.0	1.2	1.0	1.1	1.2	1.1	1.3	1.1
**A_c_, RSD (%)**	1.3	1.4	1.2	1.3	1.9	1.8	1.9	2.0
***Intermediate precision*** **^k^**								
**t, RSD (%)**	1.2	1.1	1.2	1.1	1.1	1.4	1.3	1.5
**A_c_, RSD (%)**	1.9	2.0	1.6	1.7	2.1	2.5	1.5	2.2
**LOD ^l^**	0.4 mg L^−1^		0.4 mg L^−1^		0.3 mg L^−1^		0.3 mg L^−1^	
**LOQ ^m^**	1.4 mg L^−1^		1.4 mg L^−1^		0.8 mg L^−1^		0.9 mg L^−1^	

A_c_: corrected peak area. ^a^ Nine standard solutions at different concentration levels injected in triplicate for carfentrazone-ethyl and carfentrazone. ^b^ Addition of five known amounts of carfentrazone-ethyl standard racemate to commercial herbicide formulation with a constant concentration of carfentrazone-ethyl racemate (30 mg L^−1^ according to the label). ^c^ *p*-value of t-test (ANOVA) >0.05 at a confidence level of 95% demonstrated the absence of matrix interferences. ^d^ Evaluated as the mean recovery obtained from six replicates of the commercial herbicide formulation containing 30 mg L^−1^ carfentrazone-ethyl racemate (according to the label) spiked with 10 mg L^−1^ carfentrazone-ethyl standard racemate (recovery ^1^) and other six replicates of the commercial herbicide formulation spiked with 60 mg L^−1^ carfentrazone-ethyl standard racemate (recovery ^2^). ^e^ Addition of seven known amounts of carfentrazone-ethyl and carfentrazone standard racemate to 1 g of cleaned sand sample. ^f^ Evaluated as the mean recovery obtained from fourteen replicates from cleaned sand samples spiked at different racemate concentration levels (seven extracts from 0.6 to 80 mg L^−1^ carfentrazone-ethyl and seven extracts from 0.6 to 100 mg L^−1^ carfentrazone), each one injected in triplicate. ^g^ Addition of seven known amounts of carfentrazone-ethyl and carfentrazone standard racemate to 1 g of soil sample extract and soil sample, respectively. ^h^ Evaluated as the mean recovery obtained from twelve replicates from soil samples spiked at different racemate concentration levels (six soil extracts from 0.6 to 80 mg L^−^^1^ for carfentrazone-ethyl and six soil samples from 0.6 to 80 mg L^−1^ for carfentrazone), each one injected in triplicate. ^i^ Six repeated injections (*n* = 6) of carfentrazone-ethyl and carfentrazone standard racemate solutions at two concentration levels (10 mg L^−1^ and 60 mg L^−1^). ^j^ Three replicates injected three times each (*n* = 9) on the same day of each standard racemate solutions, carfentrazone-ethyl and carfentrazone (10 mg L^−1^ and 60 mg L^−1^). ^k^ Three replicates injected each in triplicate (*n* = 9) during three consecutive days of each standard racemate solutions, carfentrazone-ethyl and carfentrazone (10 mg L^−1^ and 60 mg L^−1^). ^l^ Experimentally determined as the concentration yielding a S/N ratio of 3. ^m^ Experimentally determined as the concentration yielding a S/N ratio of 10.

## Data Availability

Not applicable.

## Supplementary Materials

Figure S1 Degradation study of carfentrazone-ethyl and carfentrazone in cleaned sand samples. Recovery (%) obtained for (A) carfentrazone-ethyl and (B) carfentrazone after zero, one, three, four, and seven days of incubation. Experimental conditions as in Figure 5. Figure S2 Degradation study of carfentrazone-ethyl and carfentrazone in soil samples. Recovery (%) obtained for (A) carfentrazone-ethyl and (B) carfentrazone after zero, one, three, four, and seven days of incubation. Experimental conditions as in Figure 5. Table S1. Analysis time, resolutions between consecutive peaks, and enantiomer peak areas for carfentrazone-ethyl and carfentrazone under different experimental conditions.

## Sample Availability

Samples of the compounds are not available from the authors.
