# Supplementary material for: Simultaneous Enantiomeric Separation of Carfentrazone-Ethyl Herbicide and Its Hydrolysis Metabolite Carfentrazone by Cyclodextrin Electrokinetic Chromatography. Analysis of Agrochemical Products and a Degradation Study
Source: Molecules. 2021 Sep 2;26(17):5350. doi: 10.3390/molecules26175350 (PMC8433761; doi:10.3390/molecules26175350)
Supplement: Supplementary file 1 [file molecules-26-05350-s001.zip › molecules-1350922-supplementary.pdf]

SIMULTANEOUS ENANTIOMERIC SEPARATION OF CARFENTRAZONE-ETHYL HERBICIDE AND ITS HYDROLYSIS METABOLITE CARFENTRAZONE BY CYCLODEXTRIN ELECTROKINETIC CHROMATOGRAPHY. ANALYSIS OF AGROCHEMICAL PRODUCTS AND A DEGRADATION STUDY

Laura García-Cansino[1], María Ángeles García[1,2], María Luisa Marina[1,2]*.

[1] Universidad de Alcalá. Departamento de Química Analítica, Química Física e Ingeniería Química. Ctra. Madrid-Barcelona Km. 33.600, 28871 Alcalá de Henares (Madrid), Spain.; laura.garciacansino@uah.es (L.G.-C.); angeles.garcia@uah.es (M. A. G.); mluisa.marina@uah.es (M.L.M.)

[2] Universidad de Alcalá. Instituto de Investigación Química Andrés M. del Río. Ctra. Madrid-Barcelona Km. 33.600, 28871 Alcalá de Henares (Madrid), Spain.; angeles.garcia@uah.es (M.A.G.); mluisa.marina@uah.es (M.L.M.)

* Correspondence: mluisa.marina@uah.es; Tel.: +34 918854935; Fax: +34 91 8854971 (Universidad de Alcalá. Departamento de Química Analítica, Química Física e Ingeniería Química. Ctra. Madrid-Barcelona Km. 33.600, 28871 Alcalá de Henares, Madrid, Spain)

Figures

Figure S1. Degradation study of carfentrazone-ethyl and carfentrazone in cleaned sand samples. Recovery (%) obtained for (**A**) carfentrazone-ethyl and (**B**) carfentrazone after 0, 1, 3, 4 and 7 days of incubation. Experimental conditions as in Figure 5.

Figure S2. Degradation study of carfentrazone-ethyl and carfentrazone in soil samples. Recovery (%) obtained for (**A**) carfentrazone-ethyl and (**B**) carfentrazone after 0, 1, 3, 4 and 7 days of incubation. Experimental conditions as in Figure 5.

Figure S1. Degradation study of carfentrazone-ethyl and carfentrazone in cleaned sand samples. Recovery (%) obtained for (A) carfentrazone-ethyl and (B) carfentrazone after zero, one, three, four, and seven days of incubation. Experimental conditions as in Figure 5.

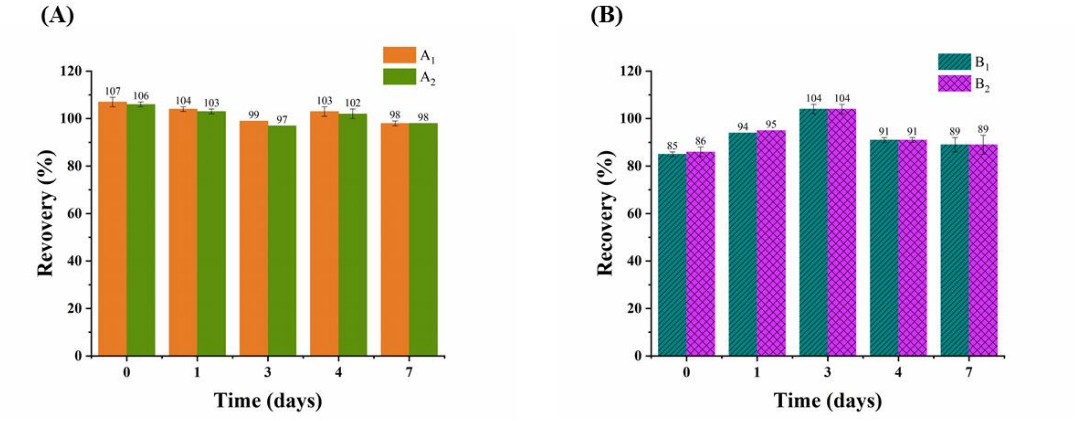

Figure S2. Degradation study of carfentrazone-ethyl and carfentrazone in soil samples. Recovery (%) obtained for (A) carfentrazone-ethyl and (B) carfentrazone after zero, one, three, four, and seven days of incubation. Experimental conditions as in Figure 5.

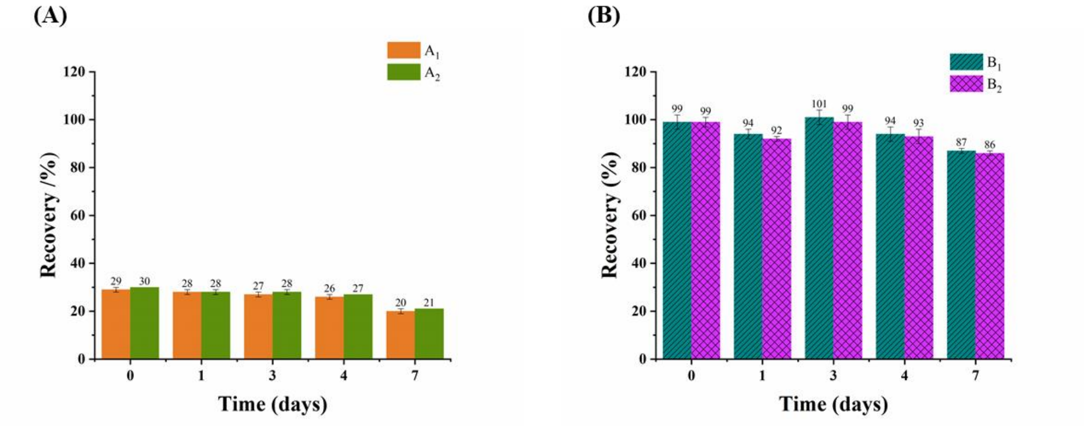

Table S1. Analysis time, resolutions between consecutive peaks, and enantiomer peak areas for carfentrazone-ethyl and carfentrazone under different experimental conditions.

**Variable**		**Analysis time (min)**	**Peak areas**				**Resolution**		
			**A_1**	**A_2**	**B_1**	**B_2**	**A_1-A_2**	**A_2-B_1**	**B_1-B_2**
**CD concentration (% (w/v))** *20 °C / −20kV / 100 mM acetate buffer pH 5*	1.0	18.40	9.8	10.1	10.2	10.2	9.33	5.22	4.84
	1.5	16.30	6.9	7.1	8.3	8.6	8.51	8.48	6.13
	2.0	15.10	5.7	5.9	6.7	6.9	7.28	6.00	5.99
	**2.5**	**14.80**	**5.8**	**6.0**	**7.1**	**7.2**	**6.54**	**5.56**	**6.15**
	3.0	13.60	6.9	7.0	8.1	8.4	5.38	2.24	5.58
	3.5	13.40	7.4	7.2	8.4	8.0	4.73	0.63	5.33
**Temperature (°C)** *2.5 % (w/v) / −20kV / 100 mM acetate buffer pH 5*	20	14.80	5.8	6.0	7.1	7.2	6.54	5.56	6.15
	25	14.20	6.0	6.1	7.5	7.8	6.94	7.04	5.02
	**30**	**12.30**	**7.1**	**7.1**	**8.7**	**9.0**	**5.72**	**5.66**	**5.29**
**Buffer concentration (mM)** *2.5 % (w/v) / 30 ºC /−20kV*	**25**	**10.90**	**7.1**	**7.1**	**8.5**	**8.8**	**5.32**	**10.15**	**5.09**
	50	12.10	7.7	7.8	9.0	9.2	5.83	7.16	4.14
	100	12.30	7.1	7.1	8.7	9.0	5.72	5.66	5.29
**Voltage (kV)** *2.5 % (w/v) / 30 ºC / 25 mM acetate buffer pH 5*	−20	10.90	7.1	7.1	8.5	8.8	5.32	10.15	5.09
	−25	8.40	5.2	5.1	6.2	6.3	4.92	10.56	5.01
	**−30**	**6.80**	**3.6**	**3.7**	**5.2**	**5.3**	**4.99**	**11.38**	**5.11**
